# Supplementary material for: Biocompatibility of ABS and PLA Polymers with Dental Pulp Stem Cells Enhance Their Potential Biomedical Applications
Source: Polymers (Basel). 2023 Dec 6;15(24):4629. doi: 10.3390/polym15244629 (PMC10747830; doi:10.3390/polym15244629)
Supplement: Supplementary file 1 [file polymers-15-04629-s001.zip › polymers-2719117-supplementary.pdf]

Supplementary Materials:

Table S1. This table presents information on the donor's age, sex, number of days and cell concentration at the time of the first passage.

Samples	A	B	C	Average
Age	20	24	15	19,6
Gender	feminine	feminine	male	-
Cultivation days for first pass	25	17	10	17
Cell concentration in the first pass	$1.95x10^6$	$4.8x10^6$	$4.85x10^6$	$3.86x10^6$

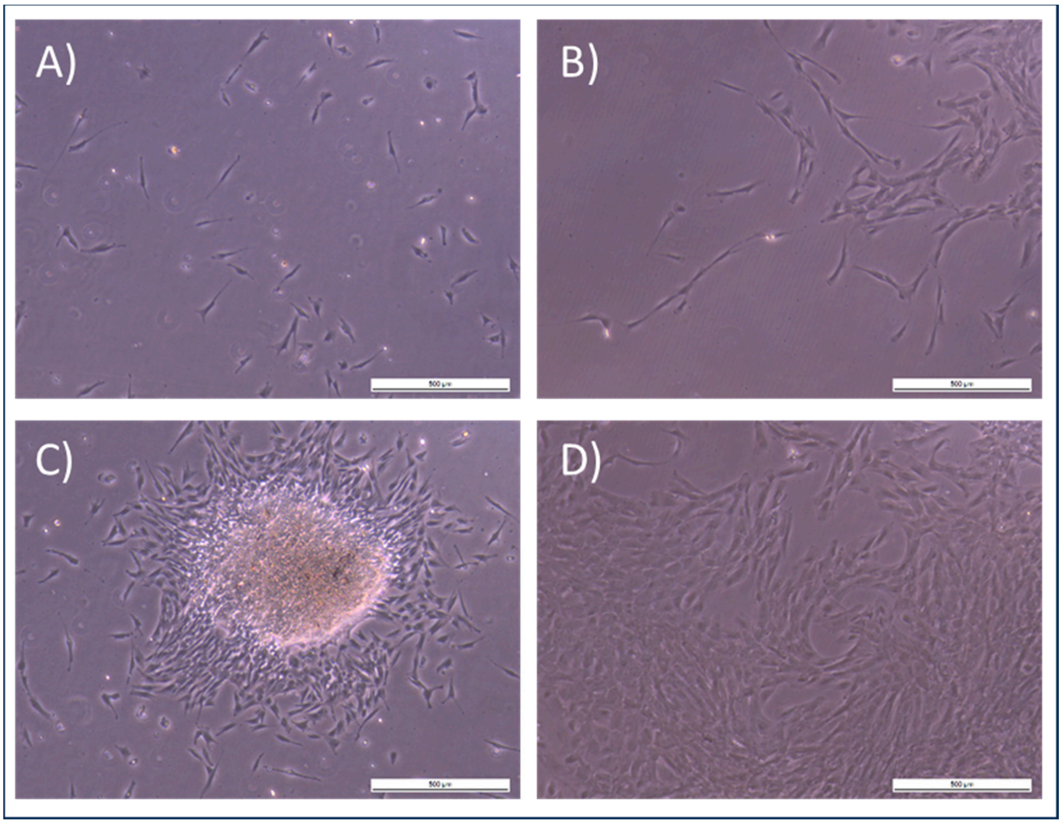

Figure S1. Representative photomicrographs of cell culture, adherence to plastic and fibroblastoid morphology were observed throughout cell culture. A) Beginning of cell adhesion. B) Cells adhered to the culture flask and beginning of cell proliferation (Day 5). C) Proliferation of cells in colonies (Day 10). D) Aspect of cell culture with confluence greater than 80% (Day 17), when the passages were carried out. (50X magnification, scale bar: 500 µm).

Table S2. Comparison by the Friedman test of the median and quartile obtained in the bioluminescence analyses comparing the days of intragroup analysis.

		**Median**	**Q25**	**Q75**	*p*
Group DPSC	Day 2	5.70E6	4.57E6	2.59E7	<0.001
	Day 3	1.81E6	1.26E6	1.27E7	
	Day 4	6.39E6	3.12E6	1.78E7	
	Day 5	1.29E7	6.24E6	3.04E7	
	Day 6	3.34E7	1.17E7	8.45E7	
	Day 7	7.17E7	2.45E7	1.38E8	
Group DPSC+ ABS	Day 2	1.12E7	9.54E6	1.32E7	<0.001
	Day 3	2.40E6	1.44E6	5.66E6	
	Day 4	5.65E6	2.43E6	7.76E6	
	Day 5	1.13E7	5.07E6	1.42E7	
	Day 6	2.83E7	8.91E6	3.64E7	
	Day 7	5.84E7	1.80E7	9.72E7	
Group DPSC+ PLA	Day 2	1.48E7	1.08E7	1.94E7	<0.001
	Day 3	2.83E6	2.15E6	1.05E7	
	Day 4	6.34E6	2.14E6	1.04E7	
	Day 5	1.33E7	4.31E6	1.61E7	
	Day 6	3.51E7	8.15E6	4.06E7	
	Day 7	7.39E7	1.75E7	9.48E7	

Table S3. Comparison by the Kruskal-Wallis test of the median and quartile obtained in the bioluminescence analyses comparing the groups on different days of analysis.

	Median	Q25	Q75	*p*
**Day 2**				
DPSC	5.7E6	4.57E6	2.59E7	
DPSC+ABS	1.12E7	9.54E6	1.32E7	<0.155
DPSC+PLA	1.48E7	1.08E7	1.94E7	
**Day 3**				
DPSC	1.81E6	1.26E6	1.27E7	
DPSC+ABS	2.40E6	1.44E6	5.66E6	0.546
DPSC+PLA	2.83E6	2.15E6	1.05E7	
**Day 4**				
DPSC	6.39E6	3.12E6	1.78E7	
DPSC+ABS	5.65E6	2.43E6	7.76E6	0.713
DPSC+PLA	6.34E6	2.14E6	1.04E7	
**Day 5**				
DPSC	1.29E7	6.24E6	3.04E7	
DPSC+ABS	1.13E7	5.07E6	1.42E7	0.597
DPSC+PLA	1.33E7	4.31E6	1.61E7	
**Day 6**				
DPSC	3.34E7	1.17E7	8.45E7	
DPSC+ABS	2.83E7	8.91E6	3.64E7	0.601
DPSC+PLA	3.51E7	8.15E6	4.06E7	
**Day 7**				
DPSC	7.17E7	2.45E7	1.38E8	
DPSC+ABS	5.84E7	1.80E7	9.72E7	0.589
DPSC+PLA	7.39E7	1.75E7	9.48E7	

Table S4. Comparison by the Wilcoxon between the p values obtained from the variables on intragroup days in the bioluminescence analyses.

**Days**	*p*
Day 2 x Day 3	0.008
Day 2 x Day 4	0.051
Day 2 x Day 5	0.008
Day 2 x Day 6	0.008
Day 2 x Day 7	0.008
Day 3 x Day 4	0.008
Day 3 x Day 5	0.008
Day 3 x Day 6	0.008
Day 3 x Day 7	0.008
Day 4 x Day 5	0.008
Day 4 x Day 6	0.008
Day 4 x Day 7	0.008
Day 5 x Day 63	0.008
Day 5 x Day 7	0.008
Day 6 x Day 7	0.008

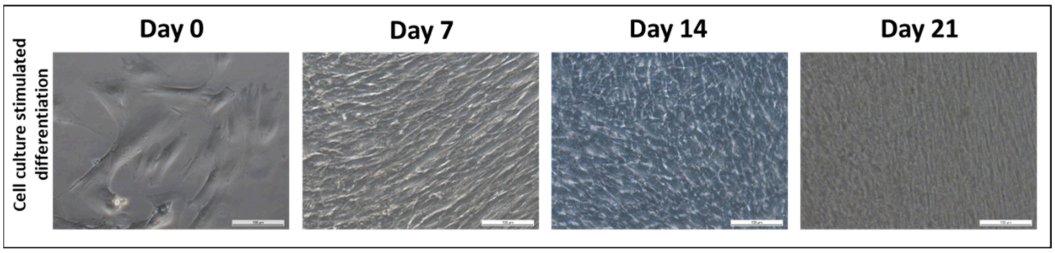

Figure S2. Photomicrographs of cell differentiation. A) Morphological monitoring of DPSC during stimulation of osteogenic differentiation (0, 7, 14 and 21 days) cultured on a coverslip (magnification 100x, scale bar: 100 µm).

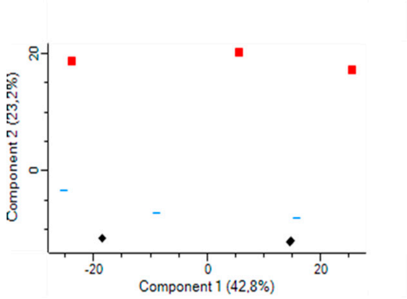

Figure S3. Principal component analysis (PCA) of the protein samples analyzed using the Perseus software. The red squares represent the control samples, the black diamonds the ABS samples and the blue horizontal lines the PLA samples. Note that two black diamonds are overlapping (n=3).

Table S5. Raw data. Proteins groups used for FunRich and Perseus analyses.
Available in: https://docs.google.com/spreadsheets/d/1NUbN9lOu--r4hJGQ6JBfMw17W5OIK_6D/edit?usp=sharing&ouid=108025814496751279025&rtpof=true&sd=true
